# A Model of Phlebitis Associated with Peripheral Intravenous Catheters in Orthopedic Inpatients

**DOI:** 10.3390/ijerph16183412

**Published:** 2019-09-14

**Authors:** Sookhee Lee, Kyunghee Kim, Ji-Su Kim

**Affiliations:** 1 Head Nurse of Department of Nursing, Kangdong Sacred Heart Hospital, 150, Seongan-ro, Gangdong-gu, Seoul 06974, Korea; kuum98@naver.com; 2 Faculty of Department of Nursing, Chung-Ang University, Seoul 06974, Korea; jisu80@cau.ac.kr

**Keywords:** infection, inpatient, intravenous injection, phlebitis, orthopedic

## Abstract

Phlebitis leads to increased discomfort for patients, longer hospital stays, and higher healthcare costs. This study aimed to identify predictive factors of peripheral phlebitis related to intravenous injection among orthopedic inpatients, develop a prediction model, and evaluate the goodness-of-fit of the prediction model. This study included 270 orthopedic patients who were hospitalized in the orthopedic ward of a general hospital. A peripheral intravenous injection-related questionnaire based on previous studies and the modified Infusion Nurses Society scale were used to collect data. Phlebitis risk factors were identified, and a prediction model was developed using the Bayesian regression model. Vein quality, contrast medium use, hygiene duration, and period of nursing clinical experience were significant based on their 95% confidence intervals. The prediction model exhibited good discrimination. The prediction model developed in this study can be used for screening high-risk patients for peripheral intravenous catheter-related phlebitis and for providing basic data for developing interventions for the prevention and management of peripheral intravenous catheter-related phlebitis.

## 1. Introduction

Intravenous injection via a peripheral intravenous catheter (PIVC) is the most common route of administering medication [1]. Approximately 60% of hospitalized patients have at least one PIVC [2], and 80.6% [3] to 86.4% [4] of hospitalized patients are administered intravenous bolus/push medication. Given the diverse utility of intravenous injections, such as administration of fluid, medications, and contrast agents required for imaging [5], their usage is predicted to increase even further. Despite this utility, numerous complications are associated with intravenous bolus/push medication administration, including phlebitis, infiltration, burning sensation, and fluid volume excess [6].

Phlebitis is characterized by inflammation of the vein wall and can be accompanied by symptoms such as edema, pain, and erythema near the catheter insertion site or along the affected vein, sometimes progressing to palpable venous cord, intense redness, tenderness, and fever [7]. Factors affecting the incidence of phlebitis can be classified into individual factors, such as sex [8], age [9], underlying health conditions such as infectious or hypertensive disease and surgery [8], and caregiver residence [10]; chemical factors, such as the osmolality of the injected drug [11], number of medications [12], type of antibiotics [13], and rate and method of drug injection [14]; mechanical factors, such as catheter dwell time, which can cause friction due to intravascular movement of the catheter [15], catheter insertion site [15,16], and catheter size [12]; and infectious factors, such as hand hygiene of health professionals [17] and nurse’s skill in administering intravenous injection [18]. However, there is little literature on phlebitis associated with PIVC, and it is less conclusive [19].

Phlebitis leads to increased discomfort for patients, longer hospital stays, and higher healthcare costs [20]. In particular, in Korea, orthopedic patients with phlebitis show a longer mean length of stay, at 11.7 d, than those without phlebitis, at 9.3 d [21]; thus, negligent phlebitis control leads to longer periods of hospitalization, which increases the suffering and economic burden for the patient. Moreover, vigorous movement of the upper body, such as during gait with a walking device, increases the pressure in the veins of the upper extremities, and movement of the catheter in the vein can also cause phlebitis; as a result, use of a crutch in orthopedic patients has been reported as a contraindication for placement of a peripherally inserted central catheter in the upper arm [22]. Therefore, phlebitis should be considered for orthopedic patients frequently using walking devices, including crutches, wheelchairs, or walkers. Furthermore, orthopedic inpatients usually undergo surgical treatment and are administered antibiotics for therapeutic (9.2%) or preventive (90.8%) purposes in Korea [23]. Antibiotics generally irritate blood vessels [18], and low pH causes chemical phlebitis [24]. However, there have not been any studies of phlebitis in orthopedic inpatients.

This study aimed to identify individual, chemical, mechanical, and infectious factors reported to be associated with PIVC-related phlebitis, to develop a prediction model for the incidence of PIVC-related phlebitis in orthopedic inpatients, and to evaluate the prediction model by a goodness-of-fit test.

## 2. Methods

### 2.1. Design

This was a prospective study using survey research to identify prediction factors for PIVC-related phlebitis in orthopedic inpatients.

### 2.2. Participants

The participants consisted of 270 orthopedic patients, aged at least 19 years old, who had been hospitalized for at least 4 days (based on the 2002 Centers for Disease Control (CDC) guidelines to replace PIVCs every 72–96 h) in an orthopedic ward at a single general hospital in Seoul (with 640 beds) and who had received intravenous therapy.

### 2.3. Data Collection

Data were collected between 1 February and 12 May 2017 by seven nurses with at least 5 years of clinical experience. The peripheral intravenous injection-related questionnaire was developed by a researcher, modified, and supplemented following review by a professor of nursing and 3 nurses with at least 10 years of clinical experience. The final questionnaire consisted of 9 questions on individual factors, 8 questions on chemical factors, 4 questions on mechanical factors, 2 questions on infectious factors, and 1 question on whether the participant developed phlebitis. The questionnaire was distributed in participants’ wards, and data collection was done by a nurse. A researcher checked the record as necessary and supplemented any missing information with electronic medical records. Herein, for personal factors, vein quality was classified into three categories (good, fair, or poor) in accordance with the Vein Assessment Tool (VAT) [25], and activity level was classified into inactive (0–1) or active (>1) using the Physical Activity in Inpatient Rehabilitation Assessment [26].

In this study, the modified Infusion Nurses Society (INS) scale [27] was used to measure phlebitis. The seven ward nurses who would monitor phlebitis were educated before the start of the study regarding the definition of phlebitis, method of filling out the peripheral intravenous injection-related questionnaire, and other precautions, after which they were asked to assess phlebitis from 10 actual photographs, and the inter-/intra-rater reliability was calculated. Using the test–retest method, the inter-rater weighted kappa was 0.955, and the intra-rater reliability-weighted kappa was 0.973. A kappa of 0.75 or better indicates good inter-rater reliability [28].

### 2.4. Ethical Considerations

This study was approved by an appropriate institutional review board (KANGDONG 2016-02-011-011). The study was conducted according to the principles of the Declaration of Helsinki. The study participation consent form included statements that participants could withdraw their participation at any time, collected data would only be used for research purposes, and participants’ anonymity would be protected. Participants provided consent voluntarily after a thorough explanation.

### 2.5. Data Analysis

Data were analyzed using the statistics program R 3.4.2 version (R core team, Auckland, New Zealand). The variables of phlebitis occurrence, by grade, and personal, chemical, mechanical, and infectious factors related to intravenous injection were analyzed using descriptive statistics (frequency and percentage, mean, and standard deviation). Differences in these variables between the phlebitis and no phlebitis groups were analyzed using the *χ*^2^ test.

The prediction model for PIVC-related phlebitis was analyzed by Bayesian regression, and the sample was generated using the Hamiltonian Monte Carlo algorithm, which is a Markov chain Monte Carlo (MCMC) method [29]. In this model, 20,000 samples were generated with repetition using the Hamiltonian Monte Carlo method, and the minimum effective sample size for each coefficient was 8275 (41.4%); hence, the coefficients with a minimum effective sample size that did not exceed 41.4% were excluded, as the generated sample was considered not to be convergent. After sample convergence diagnosis, the 20,000 selected samples were used to estimate Bayesian logistic regression coefficients, and the significance of the estimated coefficients was inspected using the 95% credible intervals (95% CIs). We identified predictive factors for PIVC-related phlebitis from the 95% CI model and calculated the effect sizes in terms of odds ratios (ORs). The fit of the prediction model was analyzed using the log-likelihood function (−2*L*(θ)), pseudo R^2^, Deviance Information Criterion (DIC), Hosmer–Lemeshow (H-L) statistic, standardized mortality ratio (SMR), and the area under the curve (AUC) from the receiver operating characteristic (ROC) curve.

## 3. Results

### 3.1. Incidence and Severity of Phlebitis Based on the Modified INS Scale

Among the 270 participants, 64.1% did not develop phlebitis, while 35.9% developed phlebitis, with most (18.1%) being grade 0+ (Table 1).

### 3.2. Association between Intravenous Injection-Related Factors and the Incidence of Phlebitis

Among personal factors, vein quality (*χ*^2^ = 17.40, *p* <0.001) and activity level (*χ*^2^ = 4.33, *p* = 0.037) were significantly correlated with phlebitis incidence. Among chemical factors, fluid therapy (*χ*^2^ = 4.85, *p* = 0.028), drugs with high osmolality (*χ*^2^ = 9.72, *p* = 0.002), use of contrast medium (*χ*^2^ = 27.46, *p* < 0.001), and method of infusion (*χ*^2^ = 10.18, *p* = 0.006) were significantly correlated with phlebitis incidence. Among mechanical factors, catheter dwell time (*χ*^2^ = 16.84, *p* = 0.001) was significantly correlated with phlebitis incidence. Among infectious factors, hand hygiene duration (*χ*^2^ = 60.02, *p* < 0.001) and period of nursing clinical experience (*χ*^2^ = 24.66, *p* < 0.001) were significantly correlated with phlebitis incidence (Table 2).

### 3.3. Predictive Factors for PIVC-Related Phlebitis in the 95% CI Model

In the 95% CI model, significant variables with a 95% posterior probability not including 0 were as follows (six categories): personal factor of vein quality (fair 95% CI: 0.095, 1.728; poor 95% CI: 0.880, 3.420), chemical factor of use of contrast medium (no 95% CI: −3.958, −1.366), infectious factors of hand hygiene duration (≥10 to <20 s 95% CI: −2.413, −0.828; ≥20 to <30 s 95% CI: −8.363, −3.617), and period of nursing clinical experience (≥3 to <5 years 95% CI: −3.480, −0.561) (Table 3).

### 3.4. Effect Sizes of Predictive Factors for PIVC-Related Phlebitis in the 95% CI Model

The personal factor of vein quality (fair, poor) was a significant variable. Compared to good vein quality, fair vein quality (OR: 2.514, 95% CI: 1.100, 5.630) showed a 2.51-fold higher phlebitis occurrence probability, while poor vein quality (OR: 8.286, 95% CI: 2.413, 30.585) showed an 8.28-fold higher phlebitis occurrence probability. The chemical factor of use of contrast medium was a significant variable. Compared to use of contrast medium, nonuse of contrast medium (OR: 0.074, 95% CI: 0.019, 0.255) was associated with a 92.5% reduction in the phlebitis occurrence probability. Among infectious factors, hand hygiene duration (≥10 to <20 and ≥20 to <30 s) and period of nursing clinical experience (≥3 to <5 years) were significant variables. Compared to hand hygiene duration of <10 s, ≥10 to <20 s duration (OR: 0.202, 95% CI: 0.089, 0.436) was associated with a 79.7% reduction in the phlebitis occurrence probability, and ≥20 to <30 s duration (OR: 0.003, 95% CI: 0.000, 0.026) was associated with a 99.6% reduction in the phlebitis occurrence probability. Compared to a period of nursing clinical experience of <1 year, a period of ≥3 to <5 years (OR: 0.136, 95% CI: 0.030, 0.570) was associated with an 86.3% reduction in phlebitis occurrence probability (Table 4).

### 3.5. Fit of the Prediction Model for PIVC-related Phlebitis

We derived the posterior functions for the Bayesian logistic regression model based on the log-likelihood function and prior information and estimated coefficients by simulation. If the estimated coefficients maximized the log-likelihood function of the model, which indicates that they provided the most possible information, a low value for the negative log-likelihood function was obtained (−2*L*(θ)). In addition, since the log-likelihood function tends to increase with increasing sample size, the model fit should be examined using various indices of fit [30]. In this model, the value of the log-likelihood function was −94.868.

**Figure 1:**
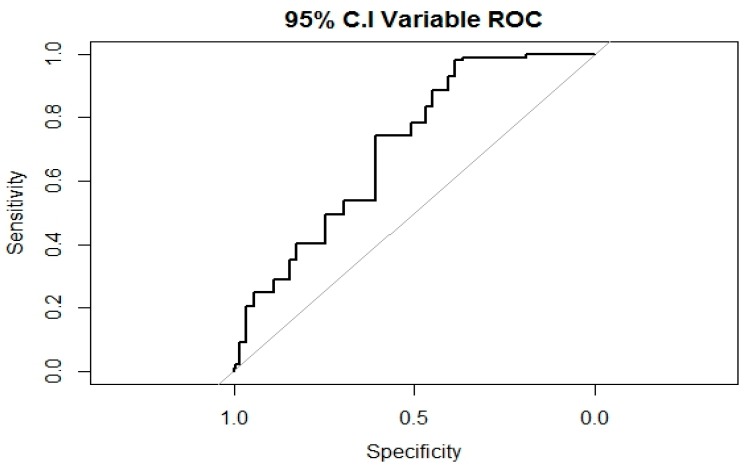
Receiver operating characteristic curve.

In a Bayesian logistic regression model, the log-likelihood function can be used to calculate pseudo R^2^, where a value closer to 1 indicates higher explanatory power of the model [31]. In our study, pseudo R^2^ was 0.462. DIC is calculated by adding the number of effective parameters in the model to a value derived from the likelihood function, which is given as the mean value of each parameter generated by MCMC from the posterior distributions. A smaller DIC value indicates a smaller difference between the predicted values and the actual values [32]. In our study, DIC was −105.339. The H-L statistic was calculated under the assumption that the model for which fit is being analyzed was similar to the observed data; a higher value for the statistic leads to the conclusion that the model differs from the observed data and is, thus, interpreted as worse fit. In particular, if the p-value for the null hypothesis H-L statistic is lower than the significance level, this shows poor fit [33]. In this study, the H-L statistic was 1.366 (*p* = 0.243). The SMR is used to examine differences between the predictions of the model and observed data, where a value of 1 shows that the mortality rates for the observed and predicted data are the same. If SMR is greater than 1, this means that there are more observed deaths than predicted deaths, and if SMR is lower than 1, this means that there are fewer observed deaths than predicted deaths [34]. In our study, SMR was 1.102. We calculated the AUC from the ROC curve of the PIVC-related prediction model (Figure 1).

The AUC can be classified into “no information” (AUC = 0.5), “poor accuracy” (0.5 < AUC ≤ 0.7), “moderate accuracy” (0.7 < AUC ≤ 0.9), “high accuracy” (0.9 < AUC < 1), and “perfect test” (AUC = 1) [35]. In our study, the AUC was 0.716.

## 4. Discussion

In this study, we used the modified INS scale, which also diagnoses patients’ subjective pain as phlebitis [27]. As a result, our sample had a phlebitis occurrence rate of 35.9%, with half of these as grade 0+, corresponding to pain at site, but no clinical symptoms. Phlebitis is diagnosed by various instruments and guidelines that include objective clinical signs and subjective symptoms reported by patients [36]; consequently, the phlebitis occurrence rates reported in previous studies differ in the range of 20% to 70% [36]. Despite their widespread use, the diagnostic performance of the various signs, symptoms, and assessment scales has not yet been rigorously evaluated [36]. If the patient immediately informs a nurse when they experience subjective symptoms and the catheter is removed, this can minimize the risk of PIVC-related complications [37], and the main symptoms that can be reported by patients is pain with discomfort. Therefore, patient pain should be prioritized in the diagnosis of phlebitis.

In the prediction model for PIVC-associated phlebitis, among personal factors, vein quality was a significant predictor. In this study, fair and poor vein qualities were associated with an increased risk of phlebitis compared to good vein quality. Veins suitable for intravenous injection are hard, bumpy, flat, soft, elastic, and engorged, while hardened blood vessels should be avoided [38]. In summary, poor-quality peripheral veins increase the risk of PIVC-related phlebitis. Checking vein quality by palpation and visual inspection can prevent adverse effects, such as phlebitis [39]. Our study also used VAT to evaluate vein quality based on palpation and visual inspection and reaffirmed that lower vein quality is associated with a high risk of PIVC-related phlebitis. Therefore, nurses should obtain information about the patient’s vein quality using VAT and use this to predict the risk of PIVC-related phlebitis.

Among chemical factors in the PIVC-related phlebitis prediction model, use of contrast medium was a significant predictor. In our study, nonuse of contrast medium was associated with a significant reduction in the risk of phlebitis compared to use of a contrast medium. Moreover, high or low pH and high osmolality of a drug or fluid can increase the risk of phlebitis [40]. Most contrast agents are hypertonic vesicants with an osmolality of 290–844 mOsm/L that can cause tissue damage, and rapid injection of contrast agents using a volumetric infusion pump can cause extravasation injury [41]. Therefore, care should be taken if the catheter is not immediately changed after injecting a contrast agent, since the high osmolality of the contrast agent and the method of injection are factors that can cause phlebitis.

Among infectious factors in the PIVC-related phlebitis prediction model, hand hygiene duration and period of nursing clinical experience, which is an indirect measure of nursing competence, were significant predictors. In healthcare, awareness of the importance of hand hygiene is constantly growing. In particular, hand hygiene, involving washing the hands with soap and water or applying disinfectant to the hands without water, is fundamental to the prevention of healthcare-associated infections when performed properly, as it eliminates contaminants from the hands [42]. Hand disinfection through appropriate methods, using either alcohol gel or soap, is clearly recommended in guidelines published by the CDC [43]. Alcohol gel is used in many healthcare institutions because of its broad-spectrum antibacterial activity, persistence, and rapid disinfection effect [42]. However, unlike hand hygiene using soap and water or antibacterial soap, the antibacterial effects of disinfection using alcohol gel differ among individuals, depending on the concentration of alcohol in the disinfectant, amount used in a single dose, and contract duration [44]. The manufacturer’s recommendations on the appropriate amount of alcohol-based disinfectant should be followed [45]. Although each formulation is different, most products recommend using an amount that takes 20–30 s to apply, rub between the hands, and dry, and hands should be dried completely after disinfection [46]. In our study, spending 10–20 or 20–30 s on hand hygiene was associated with a significant reduction in the risk of PIVC-related phlebitis compared to spending less than 10 s; this demonstrates the importance of hand hygiene before intravenous catheter placement. Recently, hand rubbing for 15 s was not inferior to 30 s in reducing bacterial counts on hands under the described experimental conditions in a previous study [44]. Many guidelines recommend hand disinfection before PIVC placement to reduce the risk of infection, but studies on the relationship between hand disinfection before PIVC placement and PIVC-related complications such as phlebitis are extremely few, and there is a lack of specific guidelines. Therefore, further studies are needed to assess the clinical significance of our findings.

In our study, the probability of PIVC-related phlebitis decreased when the period of nursing clinical experience was 3–5 or 5–7 years compared to less than 1 year. However, the probability of phlebitis actually increased when the period of nursing clinical experience was 7 years or longer. Nurses play an important role in the maintenance and surveillance of intravascular catheters and the control of infection [47]. Several studies have reported a lower risk of catheter infection for well-trained IV therapists compared to regular nurses [48]. The CDC also emphasizes the importance of nurses’ clinical experience and knowledge on reducing catheter-related bloodstream infection [15]. Catheter placement by a trained intravenous injection team has been reported to lower the incidence of phlebitis by reducing the number of vein puncture attempts [49], but few hospitals in Korea have a specialized nursing team for intravenous injections. Hence, further research is required regarding the relationship between clinical experience and PIVC-related phlebitis. In the present study, we used clinical experience as an indirect measure of nursing skill, dividing subjects into categories of “<1 year”, “≥1 to <3 years”, “≥3 to <5 years” and “≥5 to <7 years” [50], and found that 3–5 and 5–7 years of clinical experience were associated with a reduced rate of phlebitis compared to <1 year, but that ≥7 years of clinical experience was actually associated with a 2.04-fold increase in phlebitis. This is thought to be influenced by other variables because of the common practice of having the most experienced nurse place the catheter in cases where placement is difficult, such as in patients with poor vein quality or when catheter placement attempts have already failed at least twice.

Nevertheless, caution is required in interpreting the results of our study, as there were several limitations. First, because we only investigated orthopedic patients at a general hospital, there are restrictions in generalizing the predictive factors and prediction model for phlebitis. Second, because we did not classify phlebitis by severity, we were unable to identify differences in predictive factors for phlebitis of different severities. In the future, it will be important to investigate predictive factors of phlebitis according to its severity to develop a prediction model for clinical use. Third, this was single-center study, so we were unable to include certain factors as variables because they were identical across all patients, such as the mechanical factors of catheter material and fixation method (dressing) and the infectious factor of skin disinfectant before catheter insertion. Further multicenter studies that can reflect these diverse variables are warranted.

## 5. Conclusions

This study developed a PIVC-related phlebitis prediction model and identified an incidence of PIVC-related phlebitis of 35.9% of the studied cohort. In the 95% CI prediction model, we identified four significant predictive factors (six categories) for PIVC-related phlebitis, namely, vein quality (fair and poor), use of contrast agent (non-use), hand hygiene (≥10 to <20 s, ≥20 to <30 s), and nursing experience (clinical experience ≥3 to <5 years). 

The results of this study are important insofar as we used a multidimensional analysis, including individual, chemical, mechanical, and infectious factors, to investigate independent risk factors for PIVC-related phlebitis in orthopedic inpatients who underwent intravenous injections, and we developed a prediction model for PIVC-related phlebitis based on these factors. We believe that this prediction model can help improve the quality of intravenous nursing by applying this model in the screening patients at a high risk for phlebitis and help prevent and control PIVC-related phlebitis.

## Figures and Tables

**Table 1:** Incidence and severity of phlebitis based on the modified INS scale (*n* = 270).

Grade	Criteria	*n* (%)
0	No clinical symptoms	173 (64.1)
0+	Pain at site, but no clinical symptoms	49 (18.2)
1+	Erythema at access site with or without pain	29 (10.7)
2+	Pain at access site with erythema and/or edema	16 (5.9)
3+	Pain at access site with erythema and/or edema, streak formation, palpable venous cord	3 (1.1)
4+	Pain at access site with erythema and/or edema, or palpable venous cord > 1 inch, purulent drainage	0 (0)

Phlebitis defined as ≥0+ (pain); INS, Infusion Nurses Society.

**Table 2:** Patient characteristics as predictors of phlebitis (*n* = 270).

Characteristics		Categories	Occurrence of Phlebitis, *n* (%)			*χ* ^2^	*p*
			Total (*n* = 270)	Yes (*n* = 97)	No (*n* = 173)		
Individual	Sex	Male	136 (50.4)	47 (48.5)	89 (51.4)	0.12	0.730
		Female	134 (49.6)	50 (51.5)	84 (48.6)		
	Age (years)	<60	144 (53.3)	48 (49.5)	96 (55.5)	0.68	0.411
		≥60	126 (46.7)	49 (50.5)	77 (44.5)		
	Underlying conditions	Yes	122 (45.2)	46 (47.4)	76 (43.9)	0.18	0.670
		No	148 (54.8)	51 (52.6)	97 (56.1)		
	Diagnostic areas	Upper limb	98 (36.3)	42 (43.3)	56 (32.4)	5.37	0.068
		Lower limb	145 (53.7)	43 (44.3)	102 (59.0)		
		Spine	27 (10.0)	12 (12.4)	15 (8.7)		
	Underwent surgery	Yes	106 (39.3)	38 (39.2)	68 (39.3)	0.00	1.000
		No	164 (60.7)	59 (60.8)	105 (60.7)		
	Vein quality	Good	141 (52.2)	36 (37.1)	105 (60.7)	17.40	<0.001
		Fair	100 (37.0)	43 (44.3)	57 (32.9)		
		Poor	29 (10.7)	18 (18.6)	11 (6.4)		
	Caregiver residence	Yes	157 (58.1)	54 (55.7)	103 (59.5)	0.24	0.624
		No	113 (41.9)	43 (44.3)	70 (40.5)		
	Use a walking device	Yes	72 (26.7)	23 (23.8)	49 (28.3)	0.46	0.497
		No	198 (73.3)	74 (76.3)	124 (71.7)		
	Activity level	Inactive	130 (48.1)	38 (39.2)	92 (53.2)	4.33	0.037
		Active	140 (51.9)	59 (60.8)	81 (46.8)		
Chemical	Fluid therapy	Yes	229 (84.8)	89 (91.8)	140 (80.9)	4.85	0.028
		No	41 (15.2)	8 (8.2)	33 (19.1)		
	Drugs mixed with fluids	Yes	198 (73.3)	78 (80.4)	120 (69.4)	3.34	0.068
		No	72 (26.7)	19 (19.6)	53 (30.6)		
	Number of IV drugs	<3	180 (66.7)	59 (60.8)	121 (69.9)	1.93	0.164
		≥3	90 (33.3)	38 (39.2)	52 (30.1)		
	Types of antibiotics	Cephalosporin	195 (72.2)	66 (68.0)	129 (74.6)	5.91	0.116
		Penicillin	21 (7.8)	11 (11.3)	10 (5.8)		
		Others	12 (4.4)	7 (7.2)	5 (2.9)		
		Did not use	42 (15.6)	13 (13.4)	29 (16.8)		
	Drugs with high osmolality	Yes	30 (11.1)	19 (19.6)	11 (6.4)	9.72	0.002
		No	240 (88.9)	78 (80.4)	162 (93.6)		
	Use of contrast medium	Yes	37 (13.7)	28 (28.9)	9 (5.2)	27.46	<0.001
		No	233 (86.3)	69 (71.1)	164 (94.8)		
	Method of infusion	Continuous	22 (8.1)	4 (4.1)	18 (10.4)	10.18	0.006
		Intermittent	41 (15.2)	8 (8.2)	33 (19.1)		
		Continuous and intermittent	207 (76.7)	85 (87.6)	122 (70.5)		
	Total daily dose (mL)	<1000	172 (63.7)	57 (58.8)	115 (66.5)	1.28	0.257
		≥1000	98 (36.3)	40 (41.2)	58 (33.5)		
Mechanical	Catheter dwell time (h)	≤24	48 (17.8)	22 (22.7)	26 (15.0)	16.84	0.001
		25–48	122 (45.2)	54 (55.7)	68 (39.3)		
		49–72	63 (23.3)	16 (16.5)	47 (27.2)		
		73–96	37 (13.7)	5 (5.2)	32 (18.5)		
	Side of catheter insertion	Right	129 (47.8)	47 (48.5)	82 (47.4)	0.00	0.968
		Left	141 (52.2)	50 (51.5)	91 (52.6)		
	Site of catheter insertion	Hand	52 (19.3)	24 (24.7)	28 (16.2)	3.20	0.202
		Arm	211 (78.1)	70 (72.2)	141 (81.5)		
		Lower limb	7 (2.6)	3 (3.1)	4 (2.3)		
	Catheter gauge	18	123 (45.6)	48 (49.5)	75 (43.4)	0.71	0.399
		≤20	147 (54.4)	49 (50.5)	98 (56.6)		
Infectious	Hand hygiene duration (s)	<10	98 (36.3)	64 (66.0)	34 (19.7)	60.02	<0.001
		≥10 to <20	92 (34.1)	32 (33.0)	60 (34.7)		
		≥20 to <30	71 (26.3)	1 (1.0)	70 (40.5)		
		≥30	9 (3.3)	0 (0.0)	9 (5.2)		
	Period of nursing clinical experience (years)	<1 year	44 (16.3)	18 (18.6)	26 (15.0)	24.66	<0.001
		≥1 to <3	74 (27.4)	42 (43.3)	32 (18.5)		
		≥3 to <5	31 (11.5)	5 (5.2)	26 (15.0)		
		≥5 to <7	96 (35.6)	24 (24.7)	72 (41.6)		
		≥7	25 (9.3)	8 (8.2)	17 (9.8)		

**Table 3:** Peripheral phlebitis prediction model using the variables in 95% CI criteria.

Factors	Variables	Category	Reference Category	Coefficient			95% CI		Post. Prob	MCSE	ESS
				Mean	Median	SD	2.5%	97.5%			
Individual	Vein quality	Fair	Good	0.922	0.926	0.416	0.095	1.728	0.95	2.94^−3^	100.0
		Poor		2.114	2.098	0.646	0.880	3.420	0.95	4.57^−3^	99.8
Chemical	Use of contrast medium	No	Yes	−2.591	−2.566	0.659	−3.958	−1.366	0.95	4.81^−3^	93.9
Infectious	Hand hygiene (s)	≥10 to <20	<10	−1.595	−1.590	0.404	−2.413	−0.828	0.95	2.86^−3^	100.0
		≥20 to <30		−5.549	−5.407	1.205	−8.363	−3.617	0.95	1.02^−2^	69.8
		≥30		−26.158	v10.335	46.000	−168.176	−2.738	0.95	2.93	1.2
	Period of nursing clinical experience (years) _cons	≥1 to <3	<1	0.210	0.138	0.421	−0.557	1.156	NA	3.05^−3^	95.6
		≥3 to <5		−1.989	−1.965	0.739	−3.480	−0.561	0.95	5.43^−3^	92.8
		≥5 to <7		−0.775	−0.772	0.495	−1.753	0.079	NA	3.57^−3^	96.5
		≥7		0.715	0.627	0.704	−0.399	2.246	NA	5.04^−3^	97.7
				2.837	2.821	0.766	1.395	4.377	NA	NA	NA

CI, credible interval; MCSE, Monte Carlo standard error; SD, standard deviation. Post. Prob (posterior probability of coefficient) shows whether the coefficient includes 0 in the 95% or 75% CI and whether it shows an effect at 95% probability. ESS (effective sample size): in this model, the ESS for each coefficient was 41.4%, so the coefficients with an ESS lower than 41.4% were excluded from the model because they were nonconvergent.

**Table 4:** Odds ratio of using the 95% CI criteria variables of the peripheral phlebitis prediction model.

Factors	Variables	Category	Reference Category	OR			95% CI		Post. Prob
				Mean	Median	SD			
Individual	Vein quality	Fair	Good	2.514	2.525	1.155	1.100	5.630	0.95
		Poor		8.286	8.150	7.186	2.413	30.585	0.95
Chemical	Use of contrast medium	No	Yes	0.074	0.076	0.060	0.019	0.255	0.95
Infectious	Hand hygiene (s)	≥10 to <20	<10	0.202	0.203	0.088	0.089	0.436	0.95
		≥20 to <30		0.003	0.004	0.006	0.000	0.026	0.95
	period of nursing clinical experience (years) _cons	≥3 to <5	<1	0.136	0.140	0.137	0.030	0.570	0.95
				17.074	16.809	19.291	4.035	79.657	NA

CI, credible interval; OR, odds ratio; Post. prob, posterior probability of coefficient.
